# Association Between Injury History and Navicular Drop in Male Youth Soccer Players

**DOI:** 10.7759/cureus.92824

**Published:** 2025-09-21

**Authors:** Taishiro Kamasaki, Satoshi Fujimura, Yo Kichize, Takuya Suenaga, Masahide Yoshihara, Kodai Hosaka, Tasuku Shibasaki, Noriaki Maeda, Makoto Komiya, Atsuhiro Hirano

**Affiliations:** 1 Department of Rehabilitation Sciences, Faculty of Rehabilitation Sciences, Nishikyushu University, Saga, JPN; 2 Department of Rehabilitation Medicine, Medical Corporation Kouwakai Yokosuka Hospital, Saga, JPN; 3 Department of Rehabilitation Medicine, St. Mary’s Healthcare Center, Fukuoka, JPN; 4 Department of Rehabilitation Medicine, Kato Clinic, Saga, JPN; 5 Department of Rehabilitation Medicine, Hyakutake Orthopedics Hospital, Saga, JPN; 6 Department of Physical Medicine and Rehabilitation, Rehabilitation Center, Medical Corporation Kabutoyamakai Kurume Rehabilitation Hospital, Fukuoka, JPN; 7 Department of Sports Rehabilitation, Graduate School of Biomedical and Health Sciences, Hiroshima University, Hiroshima, JPN; 8 Department of Physical Therapy, Niigata University of Health and Welfare, Niigata, JPN

**Keywords:** history of injury, injury prevention, male youth soccer players, navicular drop, rehabilitation, sports medicine

## Abstract

Objective

Youth soccer players are in a period of rapid physical growth, raising concerns that the synergistic effect of rapid physical growth and high training intensity may increase the risk of injury. It has been shown that navicular drop (ND) is associated with injuries in various athletes, but there are no studies that have examined the association between ND and a history of injury in youth soccer players. This study aimed to examine the association between a history of injury and ND in male youth soccer players. The study’s results offer new injury prevention strategies for male youth soccer players.

Methods

This is a cross-sectional study. The study included 63 male youth soccer players (16 ± 1 years). Participants self-reported their injury history. ND was determined by measuring the navicular bone height with a caliper and calculating the difference between sitting and standing positions. Statistical analysis was conducted using binary logistic regression, with injury history (reference number) as the dependent variable and ND as the independent variable.

Results

The no-injury group included 34 participants (age: 16 ± 1 years), while the injury group included 29 participants (age: 16 ± 1 years). Comparison of measurement items showed that the ND was significantly lower in the no-injury group than in the injury group (p = 0.006, effect size (ES) = −0.74), with no significant differences in the other variables. The analysis revealed a significant association between injury history and ND (odds ratio (OR): 13.8 (1.8-103.6), p = 0.011). This finding was also observed in Model 2, which was adjusted for propensity scores (PS) (OR: 16.7 (1.8-158.4), p = 0.014).

Conclusions

It was found that a greater ND in male youth soccer players was associated with higher odds of injury history. To prevent injury, evaluating ND and implementing interventions to maintain and improve it was recommended.

## Introduction

Soccer is one of the world’s most popular sports, with many players under 18 years old [1]. High-level youth soccer players require regular training [2], which must be high in intensity and quality. However, youth soccer players are in a period of rapid physical growth, raising concerns that the synergistic effect of rapid physical growth and high training intensity may increase the risk of injury [3].

Studies have shown that soccer players most frequently sustain injuries to the lower limbs and lower back [4,5]. These injuries significantly impact both individual and team performance. For example, soccer players with a history of injury exhibit poorer single-leg countermovement jumps compared to those without a history of injury [6]. Additionally, soccer players who have suffered anterior cruciate ligament (ACL) injuries have been observed to experience a decrease in both playing time and the number of goals scored during the season [7]. Injuries not only impact an individual’s performance and grades but also the team’s success. Previous research has reported a correlation between lower injury rates and better team performance [8]. Therefore, preventing injuries is essential for both individual and team success. Preventing injuries in youth soccer players, a key age group that constitutes a significant portion of the playing population, is essential for enhancing overall performance.

Navicular drop (ND) is associated with injuries in amateur rugby players [9]. Moreover, ND is a contributing factor in predicting the occurrence of medial tibial stress syndrome in high school runners [10]. Interestingly, a study on individuals with a history of ACL injury suggested that significant downward displacement of the navicular bone and associated subtalar joint pronation may contribute to ACL injury [11]. Against this background, we hypothesized that a history of injury is associated with ND in soccer players. In soccer players, who often suffer lower limb and lower back injuries [4,5], clarifying the association between the history of injury and ND could support the selection of interventions aimed at maintaining or improving ND as a preventive strategy. However, since no studies have investigated the association between the history of injury and ND in soccer players, the results of our hypothesis remain unknown.

Therefore, this study aimed to examine the association between ND and lower limb and lower back injuries in male youth soccer players. We hypothesize that a history of injury is associated with ND. The results of this study will contribute to the prevention of injuries in male youth soccer players. Specifically, this study highlights the significance of evaluating ND and suggests the need for interventions to maintain and improve it.

## Materials and methods

Study design and setting

This cross-sectional study was conducted during a medical checkup on October 1, 2024.

Participants

The participants were male youth soccer players from Japan. The participants in this study belonged to a high school soccer team, one of the strongest schools in the prefecture, competing for the championship. This team typically trains and competes six days a week, with one day off. Participants were recruited through word of mouth. However, since this medical checkup aimed at preventing injuries and improving performance, all players, except those who were injured, were expected to participate. All evaluations in this medical checkup were conducted by licensed and experienced physiotherapists and occupational therapists. All measurements were conducted on the high school’s dirt playing field and in the indoor training room. The inclusion criteria included male youth soccer players who were members of a high school soccer team. The exclusion criteria included players who were injured and could not be measured, as well as those with missing data. Participants in the medical checkup were fully informed about the research’s content and purpose. Their consent and cooperation were obtained after they had thoroughly understood the information. We explained that participation in this study was voluntary and that refusing to participate would not result in any disadvantage. In particular, we emphasized that non-participation would not affect player selection. Informed consent was obtained from all participants. This study was approved by the Ethics Review Committee of Nishikyushu University, Saga, Japan (approval number: 23LAH37, approval date: February 6, 2024).

Basic data

The participants’ basic data, including age, practice time (hours per week), competition history, position, height, weight, and body mass index, were recorded. Practice time includes time spent practicing individually outside of team practice time. The positions were determined based on the ones they often play in games. The history of injury was ascertained by asking about any lower limb injuries in the past year. In this study, we operationally defined a history of injury as cases where a player was unable to play for more than one day due to injury.

Body composition

Body composition was measured using a body composition analyzer (InBody 470, InBody Japan Co., Ltd., Tokyo, Japan) based on bioelectrical impedance analysis (BIA). The participants stood barefoot on the machine with their heels touching the electrodes and held the electrodes in their hands. Body fat percentage, limb and trunk muscle mass, and fat-free mass index (FFMI) were automatically calculated. Body composition assessment using the BIA method with InBody is a reliable assessment method comparable in accuracy to the dual-energy X-ray absorptiometry (DXA) method [12].

Other measurement items

ND first palpated and marked the most prominent part of the participants’ navicular bones (i.e., the navicular tuberosity). While they were seated, ND used a caliper to measure the distance between the floor and the navicular bone in millimeters. Next, while the participants were standing, the distance between the floor and the navicular bone was measured in millimeters using a caliper. After that, the ND was determined by calculating the difference between the sitting and standing measurements. When evaluating the sitting position, we confirmed that the subtalar joint was in a neutral position. Furthermore, when measuring the standing posture, participants were instructed to relax so that their weight was evenly distributed between their feet [13]. For participants with a history of lower limb injury, the ND of the injured side was used for analysis. For participants with a history of lower back injuries, determining the left and right sides was challenging, so the maximum value was used for analysis. Since ND can be influenced by fatigue, all measurements were performed as early in the day as possible.

Sprint times were measured using a photoelectric lap timer (AO-10ch, Applied Office Co., Ltd., Tokyo, Japan). The participants were asked to sprint 50 m at maximum effort, with time measured using a photoelectric cell installed every 10 m. The starting position was arbitrary, and the times for each 10 m segment were used for analysis.

Grip strength was measured using a Smedley-type digital grip strength meter (T.K.K.3364, Takei Scientific Instruments Co., Ltd., Niigata, Japan). The grip strength test was conducted in a standing position with the elbow joint extended. The measurement was taken twice, alternating between the left and right sides, and the maximum value was used for analysis. The grip strength values obtained using the Smedley grip strength meter are highly reliable [14].

The knee extension strength was measured using a handheld dynamometer (μTasF-1, Anima Co., Tokyo, Japan). The measurement was taken while the participants were seated in a chair. The upper limbs were crossed in front of the chest, and the sensors were secured to the lower leg with the attached belt. The measurement was taken twice, alternating between the left and right sides, with the maximum value used for analysis. Knee extension strength measurements using a handheld dynamometer are reported to be highly reliable [15].

Shooting speed was measured using a speed gun (SPEEDSTER V, Bushnell, Osaka City) and a ball certified by the Japan Football Association (No. 5). The ball was placed 11 m from the center of the goal line (the standard penalty kick distance), and speed was measured using a speed gun set up 2 m behind the goal line. Participants were instructed to kick the ball as hard and fast as possible toward the center of the goal. There were no restrictions on running up to kick the ball. The measurement was taken twice, and the highest value was used for analysis.

Statistical analysis

Statistical analysis was conducted by first grouping participants based on the presence or absence of a history of lower limb or lower back injury, then comparing each measurement to examine the characteristics of each group. For continuous variables, a Student’s t-test was used, while for categorical variables, Fisher’s exact probability test was applied. Effect size (ES) was calculated using Cohen’s d and Cramer’s V, respectively. Next, we performed a binary logistic regression analysis, using the presence or absence of an injury history as the dependent variable (reference: players without a reported injury history) and ND as the independent variable (crude model). Next, to adjust for confounding, we created a model by calculating propensity scores (PS) for age [16], practice time [17], competitive experience [18], position [19], and FFMI [20], then included the PS in the analysis (adjusted model). We performed a chi-squared test to assess the model’s suitability for use. The goodness of fit of the binomial logistic regression equation was assessed using the Hosmer-Lemeshow test, and the variance inflation factor (VIF) was calculated to avoid multicollinearity. The statistical significance level was set at 5% (p < 0.05). Statistical analyses were performed using IBM SPSS Statistics software, version 28.0 (IBM Corp., Armonk, NY).

## Results

Participants analyzed in this study

**Figure 1:**
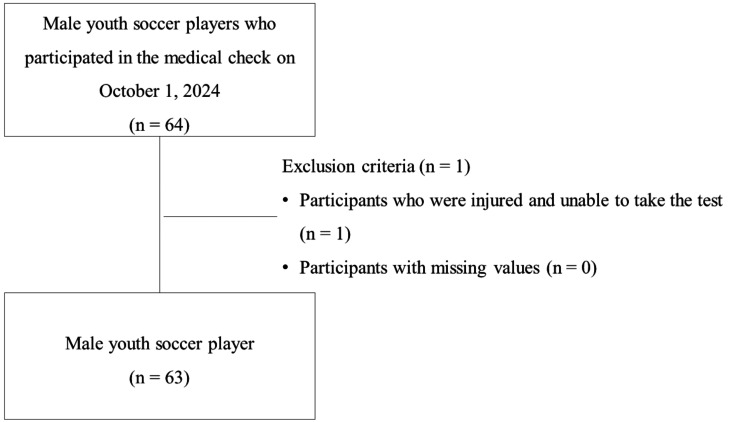
Flowchart of analysis participants

The team had 64 players. Of these, one player was excluded due to injury on the day of the medical checkup, leaving 63 participants for analysis. No participants had missing values (Figure 1). As a result, the study included 63 male youth soccer players (age: 16 ± 1 years; competitive experience: 8 ± 2 years).

Characteristics of the analyzed participants

**Figure 2:**
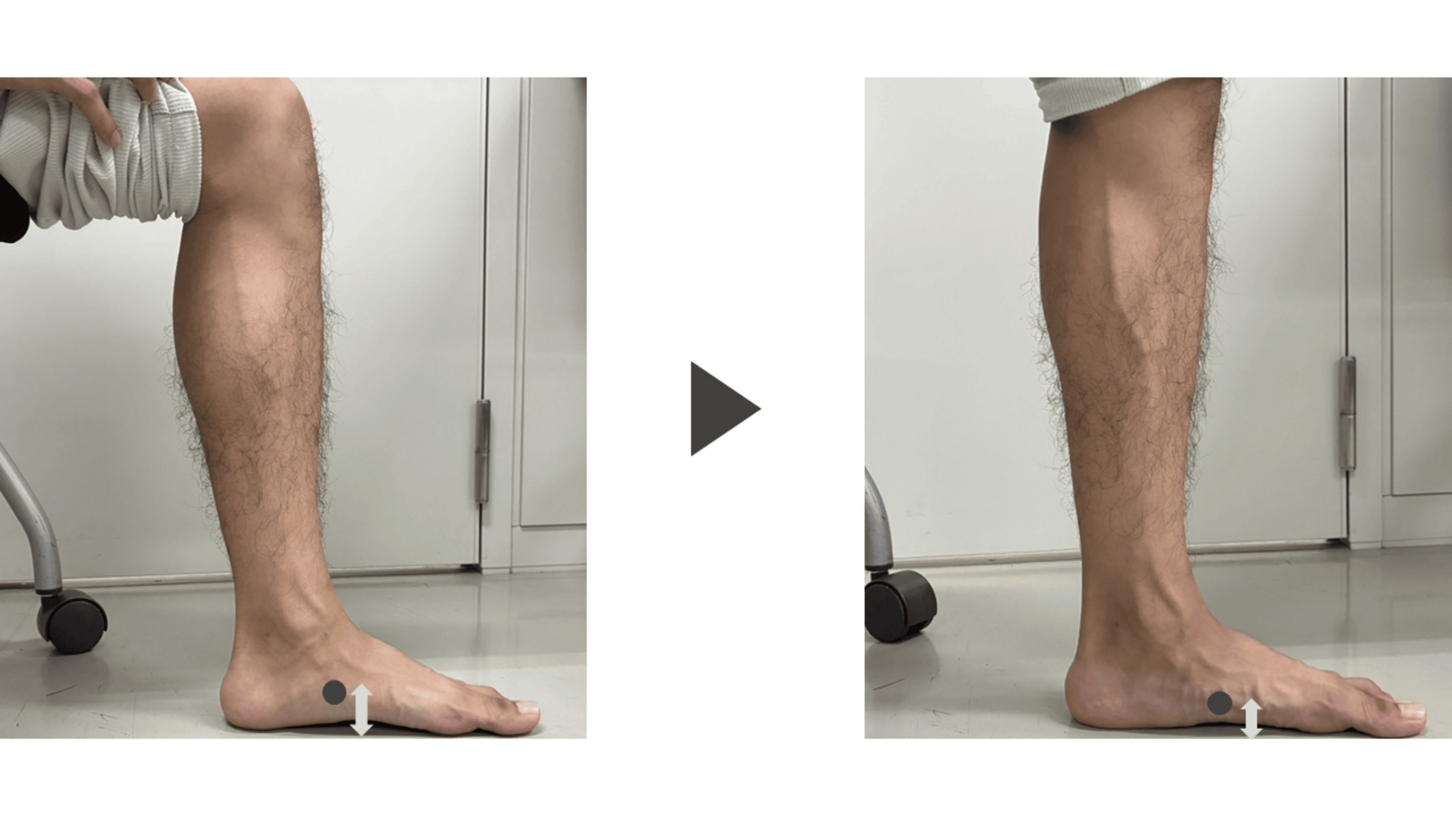
Measurement of navicular drop (ND) in seated (left) and standing (right) positions. ND was calculated as the difference in vertical distance from the floor to the navicular tuberosity between the two positions.

Table 1 shows the characteristics of the participants. The players without a reported injury history included 34 participants (age: 16 ± 1 years; competitive experience: 8 ± 2 years), while the players with a reported injury history included 29 participants (age: 16 ± 1 years; competitive experience: 9 ± 2 years). Each measurement item was compared between the two groups. As a result, the ND in the players without a reported injury history was significantly lower than that in the players with a reported injury history (p = 0.006, ES = −0.74) (Figure 2). However, no significant differences were observed in the other measured variables.

**Table 1:** Characteristics of participants in the analysis Mean ± SD, number (%); ^a^ Student’s t-test; ^b^ Fisher’s exact probability test; ^*^ Cohen's d; ^†^ Cramer's V p-values are derived from independent samples t-tests for continuous variables and Fisher’s exact tests for categorical variables. Test statistics are reported as t-values (independent samples t-test) for continuous variables and Fisher’s exact values for categorical variables. GK: goalkeeper; DF: defender; MF: midfielder; FW: forward; BMI: body mass index; BFP: body fat percentage; SMM: skeletal muscle mass; Rt: right; Lt: left; FFMI: fat-free mass index; ND: navicular drop test; 95% CI: 95% confidence interval; ES: effect size

Variables	Measurement unit	Overall (n = 63)	Players without a reported injury history (n = 34)	Players with a reported injury history (n = 29)	Test statistic	p-value	ES	95% CI (lower)	95% CI (upper)
Age	years	16 ± 1	16 ± 1	16 ± 1	-1.68	0.098 ^a^	-0.43 ^*^	-0.92	0.08
Practice time	hours/week	15 ± 3	15 ± 3	14 ± 4	0.69	0.490 ^a^	0.22 ^*^	-0.28	0.72
Competitive history	years	8 ± 2	8 ± 2	9 ± 2	-0.99	0.325 ^a^	-0.25 ^*^	-0.75	0.25
Position									
GK	n (%)	7 (11%)	6 (18%)	1 (3%)	3.28	0.352 ^b^	0.23 ^†^	0.12	0.46
DF	n (%)	21 (33%)	10 (29%)	11 (38%)					
MF	n (%)	26 (42%)	13 (38%)	13 (45%)					
FW	n (%)	9 (14%)	5 (15%)	4 (14%)					
Height	cm	169.4 ± 6.2	169.0 ± 7.0	169.8 ± 5.2	-0.45	0.653 ^a^	-0.11 ^*^	-0.61	0.38
Weight	kg	57.9 ± 6.5	57.6 ± 6.6	58.4 ± 6.4	-0.48	0.633 ^a^	-0.12 ^*^	-0.62	0.38
BMI	kg/m^2^	20.2 ± 2.1	20.2 ± 2.1	20.2 ± 2.2	0.41	0.825 ^a^	-0.06 ^*^	-0.55	0.44
BFP	%	12.6 ± 3.8	12.9 ± 3.9	12.2 ± 3.6	0.81	0.423 ^a^	0.20 ^*^	-0.29	0.70
SMM									
Rt upper	kg	2.5 ± 0.4	2.5 ± 0.4	2.6 ± 0.4	-0.67	0.506 ^a^	-0.17 ^*^	-0.67	0.33
Lt upper	kg	2.5 ± 0.4	2.5 ± 0.4	2.5 ± 0.4	-0.45	0.654 ^a^	-0.11 ^*^	-0.61	0.38
Rt lower	kg	8.5 ± 1.0	8.4 ± 1.1	8.6 ± 0.8	-0.86	0.392 ^a^	-0.22 ^*^	-0.71	0.28
Lt lower	kg	8.4 ± 0.9	8.3 ± 1.1	8.5 ± 0.8	-0.66	0.511 ^a^	-0.17 ^*^	-0.66	0.33
Trunk	kg	21.4 ± 2.3	21.3 ± 2.4	21.6 ± 2.1	-0.58	0.567 ^a^	-0.15 ^*^	-0.64	0.35
FFMI	kg/m^2^	17.6 ± 1.4	17.5 ± 1.3	17.8 ± 1.6	-0.80	0.426 ^a^	-0.20 ^*^	-0.70	0.30
ND	mm	6.0 ± 3.0	5.0 ± 3.0	8.0 ± 3.0	-2.86	0.006 ^a^	-0.74 ^*^	-1.26	-0.21
Sprint time									
10 m	seconds	2.48 ± 0.11	2.48 ± 0.12	2.47 ± 0.10	0.54	0.594 ^a^	0.14 ^*^	-0.38	0.66
20 m	seconds	3.87 ± 0.15	3.89 ± 0.16	3.84 ± 0.13	1.19	0.238 ^a^	0.32 ^*^	-0.20	0.84
30 m	seconds	5.17 ± 0.22	5.20 ± 0.24	5.13 ± 0.20	1.27	0.209 ^a^	0.34 ^*^	-0.19	0.86
40 m	seconds	6.44 ± 0.30	6.48 ± 0.32	6.37 ± 0.26	1.35	0.181 ^a^	0.36 ^*^	-0.17	0.88
50 m	seconds	7.74 ± 0.38	7.80 ± 0.40	7.67 ± 0.35	1.26	0.213 ^a^	0.33 ^*^	-0.17	0.86
Grip strength	kg	37.7 ± 5.4	35.5 ± 5.4	38.2 ± 5.8	-0.69	0.493 ^a^	-0.17 ^*^	-0.67	0.32
Knee extension strength	kg	36.9 ± 7.4	36.3 ± 7.5	35.4 ± 7.4	-0.72	0.475 ^a^	-0.19 ^*^	-0.69	0.32
Shooting speed	km/h	91.3 ± 6.2	90.4 ± 6.8	92.6 ± 5.2	-1.32	0.192 ^a^	-0.35 ^*^	-0.88	0.18

Association between the history of injury and ND

Table 2 shows the results of a binomial logistic regression analysis examining the association between the players with a reported injury history and ND. The binomial logistic regression analysis (Model 1), with the history of injury as the dependent variable and ND as the independent variable, showed a significant association between the history of injury and ND (odds ratio (OR) 13.8 (1.8-103.6), p = 0.011). In Model 2, PS was calculated from the variables considered covariates and was included to adjust for confounding. As a result, Model 2 also showed a significant association between injury history and ND (OR 16.7 (1.8-158.4), p = 0.014). In Model 2, the chi-squared tests had a p-value of 0.002, the Hosmer-Lemeshow test had a p-value of 0.574, and the discriminant accuracy rate was 63.3%. None of the independent variables had a VIF of ≥ 5.

**Table 2:** Association between history of injury and ND Binomial logistic regression analysis; dependent variable: players without a reported injury history (ref) Model 1: χ-squared tests p = 0.006, Hosmer–Lemeshow test p = 0.869, judgmental success rate 67.2%; Model 2: χ-squared tests p = 0.002, Hosmer–Lemeshow test p = 0.574, judgmental success rate 63.3%. Model 2 adjusted for confounding by including the propensity score as a covariate. Propensity scores were calculated based on age, practice time, competitive history, position, and FFMI. ND: navicular drop test; OR: odds ratio; 95% CI: 95% confidence interval; VIF: variance inflation factor; FFMI: fat-free mass index

Variable		Partial regression coefficient	OR	95% CI (lower)	95% CI (upper)	p-value	VIF
Crude Model (Model 1)	ND	2.6	13.8	1.8	103.6	0.011	―
Adjust Model (Model 2)	ND	2.8	16.7	1.8	158.4	0.014	1.05

## Discussion

The purpose of this study was to examine the association between a history of lower limb and lower back injuries and ND in male youth soccer players. The analysis revealed a significant association between the history of injury and ND.

The variables were compared between the noninjured and injured groups. As a result, a significant difference was found only in the ND. Specifically, the ND in the noninjured group was significantly lower than that in the injured group. In other words, the injured group had greater flexibility in the medial longitudinal arch (MLA) than the noninjured group. Previous research has shown that participants with a history of ACL injury have greater ND and better MLA flexibility than those without an ACL injury [11]. In addition, participants with medial tibial stress syndrome exhibited greater ND and better MLA flexibility than those without the condition [21]. Although these previous studies focused on different sports, they exhibit similar trends, supporting the validity of the findings of this study. The average ND of the participants in this study was 5.0 ± 3.0 mm for the non-injured group and 8.0 ± 3.0 mm for the injured group. In general, ND is considered indicative of excessive MLA flexibility when measuring 10 mm or more [22]. The ND of the participants in this study was less than 10 mm in both groups. Nevertheless, the significant differences between the groups are noteworthy. Further verification is needed to determine the ND values that indicate the likelihood of injury in male youth soccer players.

The binomial logistic regression analysis results showed an association between a history of injury and ND. This result remained consistent even in the model where confounding was adjusted by introducing PS. The adjusted model had an OR of 16.7. In other words, the odds of having a history of injury increase 16.7 times for every 1-mm increase in ND. A large ND leads to excessive foot pronation, which subsequently lowers the MLA and causes medial rotation of the tibia [23]. When this alignment occurs, stress on the ACL increases, elevating the risk of damage [24]. Furthermore, an increase in ND has been shown to reduce the function of the gastrocnemius muscle, which prevents ankle joint entropion and is a risk factor for lateral ankle sprains [25]. Interestingly, an increase in ND was also associated with a decline in lower back function. When the ND increases and the foot pronates, pelvic tilting and the resulting functional scoliosis may lead to a decline in trunk muscle function [26]. A decrease in trunk muscle function has been shown to reduce trunk stability, increasing the risk of musculoskeletal injuries and making it an important factor to consider [27]. Excessive increases in ND can negatively impact the alignment and function of the lower limbs and lower back, increasing the risk of injury. In this study, we were unable to investigate the alignment of the lower limbs and trunk affected by ND. However, it is possible that participants had a history of injury due to the negative impact of a large ND on the lower limbs and trunk. This remains a hypothesis. Further research is needed, but evaluating and addressing ND may help prevent injuries in male youth soccer players.

A key strength of this study is that it was the first to establish an association between a history of injury and ND in male youth soccer players. These results are significant for injury prevention in youth soccer players, who are in a period of rapid physical growth.

However, this study has some limitations that should be considered. First, the sample size is small. Furthermore, as the study only targeted one team, the possibility of sampling bias cannot be ruled out. In the future, recruiting participants from multiple teams will be necessary. Second, the severity and circumstances of the injury were not examined in detail. It will also be important to consider whether the injury was caused by contact or noncontact factors. More detailed research is needed in the future. Third, the regression models may be statistically fragile; the wide confidence intervals and the relatively small number of injury cases raise concerns about overfitting and imprecision, which should temper the interpretation of the odds ratios. Fourth, injury history was assessed through self-report and defined broadly (“unable to play for more than one day”), which may have introduced recall bias and misclassification, and the absence of clinical verification further limits the interpretability of the outcome. Finally, as this is a cross-sectional study, causal relationships cannot be determined. The cross-sectional nature precludes causal inference and temporal ordering; prospective cohort and interventional studies are needed to test whether modifying ND influences injury risk. Despite these limitations, this study provides significant findings by clarifying the association between the history of injury and ND in male youth soccer players. We believe that it will be valuable in developing future injury prevention strategies, and the results should be interpreted as exploratory and hypothesis-generating.

## Conclusions

This study demonstrated a significant association between ND and a history of lower limb and lower back injuries in male youth soccer players. ND, a simple and noninvasive measure, may serve as a practical indicator for identifying athletes at elevated risk of injury. Recognizing this relationship underscores the value of incorporating foot alignment and functional assessment into routine screening of youth players. Preventive approaches that involve regular monitoring of ND and the implementation of targeted interventions to maintain or enhance foot stability may help reduce injury risk. While further prospective studies are required to establish causality, our findings provide important insights for coaches, clinicians, and sports scientists committed to safeguarding the health and athletic development of youth soccer players.
